# Influence of Sex in the Molecular Characteristics and Outcomes of Malignant Tumors

**DOI:** 10.3389/fonc.2021.752918

**Published:** 2021-10-19

**Authors:** Jhajaira M. Araujo, Gina Rosas, Carolina Belmar-López, Luis E. Raez, Christian D. Rolfo, Luis J. Schwarz, Ulises Infante-Huaytalla, Kevin J. Paez, Luis R. García, Hober Alvarado, Fany P. Ramos, Sheyla S. Delgado-Espinoza, Jhon B. Cardenas-Farfan, Melanie Cornejo, Daniel Zanabria, Christian Colonio-Cossio, Mario Rojas-Jefferson, Joseph A. Pinto

**Affiliations:** ^1^ Centro de Investigación Básica y Traslacional, AUNA Ideas, Lima, Peru; ^2^ Escuela Profesional de Medicina Humana, Universidad Privada San Juan Bautista, Lima, Peru; ^3^ Departamento de Patología, Instituto Nacional de Enfermedades Neoplásicas, Lima, Peru; ^4^ Departamento de Genómica, Oncogenomics, Lima, Peru; ^5^ Memorial Cancer Institute/Memorial Health Care System, Florida International University (FIU), Pembroke Pines, FL, United States; ^6^ Center for Thoracic Oncology/Tisch Cancer Institute, Mount Sinai, New York, NY, United States; ^7^ Escuela Profesional de Medicina Humana-Filial Ica, Universidad Privada San Juan Bautista, Ica, Peru; ^8^ Facultad de Ciencias Biológicas, Universidad Nacional San Luis Gonzaga de Ica, Ica, Peru; ^9^ Departamento de Patología, AUNA, Lima, Peru; ^10^ División de Riesgos, Oncosalud-AUNA, Lima, Peru

**Keywords:** survival, actionable mutations, sex, GSEA, immune gene sets

## Abstract

**Background:**

Sex is frequently underestimated as a prognostic biomarker in cancer. In this study, we evaluated a large cohort of patients and public datasets to determine the influence of sex on clinical outcomes, mutational status, and activation of immune pathways in different types of cancer.

**Methods:**

A cohort of 13,619 Oncosalud-affiliated patients bearing sex-unrelated cancers was followed over a 20-year period. Hazard ratios (HRs) for death were estimated for female *vs*. male patients for each cancer type and then pooled in a meta-analysis to obtain an overall HR. In addition, the mutational status of the main actionable genes in melanoma (MEL), colorectal cancer (CRC), and lung cancer was compared between sexes. Finally, a gene set enrichment analysis (GSEA) of publicly available data was conducted, to assess differences in immune processes between sexes in MEL, gastric adenocarcinoma (GC), head and neck cancer (HNC), colon cancer (CC), liver cancer (LC), pancreatic cancer (PC), thyroid cancer (TC), and clear renal cell carcinoma (CCRCC).

**Results:**

Overall, women had a decreased risk of death (HR = 0.73, CI95: 8%–42%), with improved overall survival (OS) in HNC, leukemia, lung cancer, lymphoma, MEL, multiple myeloma (MM), and non-melanoma skin cancer. Regarding the analysis of actionable mutations, only differences in *EGFR* alterations were observed (27.7% for men *vs*. 34.4% for women, p = 0.035). The number of differentially activated immune processes was higher in women with HNC, LC, CC, GC, MEL, PC, and TC and included cellular processes, responses to different stimuli, immune system development, immune response activation, multiorganism processes, and localization of immune cells. Only in CCRCC was a higher activation of immune pathways observed in men.

**Conclusions:**

The study shows an improved survival rate, increased activation of immune system pathways, and an enrichment of *EGFR* alterations in female patients of our cohort. Enhancement of the immune response in female cancer patients is a phenomenon that should be further explored to improve the efficacy of immunotherapy.

## Introduction

Biomarkers play a key role in the selection of patients to be treated with specific therapies (1). However, there is still a quest to find an adequate and precise biomarker for immune checkpoint inhibitors (ICI) (2). Nowadays, there are a set of well-studied biomarkers, such as PD-1, PDL-1, CTLA-4, microsatellite instability (MSI), and tumor mutational burden (TMB), while other potential biomarkers are still under evaluation (3).

Sex, as a predictor of the efficacy of immunotherapy, has not been adequately evaluated in basic, translational, or clinical settings, because the effect of sex is masked by different distributions of clinical, pathological, and epidemiological characteristics between sexes, such as mutational burden, smoking status, and histology, among others (4). However, different studies have already shown that sex exerts an influence on innate and adaptive immunity during different pathogenic processes, the prognosis of autoimmune diseases, and the development of infections and malignancies (5, 6).

Furthermore, the effect of sex hormones on the PD1/PD-L1 pathway should be further studied since it could determine different responses to immunotherapy (7). These hormones also affect the number and function of immune cells, depending on cell type, tumor microenvironment, age, and reproductive status of the individual (8, 9). Several studies suggest that sex could influence the efficacy of immunotherapy as a single agent in non-small cell lung cancer (NSCLC), MEL, and other cancer types. However, other studies have observed that this difference is not statistically significant (10–13).

The decreased benefit from immunotherapy seen in women could be due to an enrichment of immune process activation in women in contrast to men (14). Paradoxically, when immunotherapy is combined with chemotherapy or radiotherapy, producing an increase of the inflammatory status in the tumoral microenvironment, better responses are observed in women compared to men (15–18). Besides, some genes that regulate the immune system escape the inactivation of the X-chromosome in women. Higher levels of mRNA from genes controlling immunity and therefore a dimorphism in the immune response are observed in female *vs*. male patients (5).

In this study, we evaluated sex-specific differences in clinical outcome, mutational status of the main activating genes, and activation levels of immune pathways in different cancer types.

## Methods

### Analysis of the Retrospective Cohort of Oncosalud-AUNA

We evaluated 13,619 oncological patients affiliated with Oncosalud-AUNA, treated between 2000 and 2019 for 20 different sex-unrelated types of cancer. The type of cancers, ICD codes, and number of patients are shown in **Supplementary Table S1**.

OS was estimated from the date of cancer diagnosis until the date of death or last follow-up. The follow-up for OS was conducted up to January 2020, using the National Civil Registry (RENIEC). To evaluate the influence of sex in OS, HR for death of female *vs*. male patients in each specific tumor was estimated and the proportional hazard assumption verified (**Supplementary Figure S1**). The meta-analysis using random effects was conducted through the software RevMan 5.4 (19).

### Tumor Samples for Sequencing

Paraffin-embedded tumor tissues were collected from advanced or metastatic MEL (n = 104), CRC (n = 208), and NSCLC (n = 291) patients diagnosed at Oncosalud-AUNA. Tumor cell content was assessed and ranged from 15% to 90% through examination of hematoxylin and eosin-stained slides by a pathologist. A commercial reference standard, Horizon Quantitative Multiplex Reference Standard HD200, was tested to validate the performance of next-generation sequencing (NGS) for detection of somatic mutations.

Eight formalin-fixed paraffin-embedded (FFPE) 8-μm-thick tissue sections were cut from FFPE tumor samples. DNA was extracted using the ReliaPrep FFPE gDNA Miniprep System (Promega, Madison, USA) following the manufacturer’s protocol. The DNA concentration was determined by fluorometric quantitation using Qubit 4.0 Fluorimeter with Qubit dsDNA HS Assay Kit (Invitrogen, USA).

### Next-Generation Sequencing

The chosen panel targeted single-nucleotide variants (SNVs) and insertion/deletions (indels) in the following genes: *BRAF*, *EGFR*, *KRAS*, *NRAS*, *PIK3CA*, and *TP53*. Libraries were prepared using the AmpliSeq Focus Panel and AmpliSeq Library Plus (Illumina, San Diego, USA) following the manufacturer’s protocol without modifications using a total of 10 ng input DNA per sample. Multiplex polymerase chain reaction (PCR) was performed in 20 cycles. Sequencing adapters with unique indexes (AmpliSeq CD Indexes Set A for Illumina) were ligated to the amplification products and purified using Agencourt AMPure XP beads (Beckman Coulter, CA, USA) according to the manufacturer’s instructions. Libraries with 2-nM molarities were subjected to clustering using a standard flow cell and were sequenced on the MiSeq platform (Illumina) using the MiSeq Reagent Micro Kit v2 (300 cycles).

### NGS Data Analysis

Raw data were processed automatically on the BaseSpace Sequence Hub (Illumina) and aligned to the hg19 reference genome. An average of 93.45% (87.2–99.7%) on-target reads, 95.2% (91.3–99.1%) read uniformity, and 500× average coverage were obtained per sample. The default limit of detection (LOD) was set at 5% allelic frequency (VAF).

BaseSpace Variant Interpreter (Illumina) was used to annotate and interpret genetic variants. Genetic variants were annotated in accordance with the nomenclature of the Human Genome Variation Society (HGVS). The interpretation was done using the Single Nucleotide Polymorphism Database (dbSNP, http://www.ncbi.nlm.nih.gov/projects/SNP/), ClinVar database, InSIGHT/LOVD database, and COSMIC. Integrative Genomics Viewer was applied to visualize the variants. All identified variants were checked with VarSome Clinical (Saphetor, Suiza). The interpretation about the pathogenicity of the variants followed the latest recommendations of the American College of Medical Genetics (ACMG), European Society for Medical Oncology (ESMO), Scale for Clinical Actionability of molecular Target (ESCAT), and OncoKB.

### Datasets for Transcriptomic Analysis

Eighteen datasets of eight types of cancers including CC, gastric (GC), HNC, LC, MEL, PC, CCRCC, and TC were retrieved from a public repository (https://www.ncbi.nlm.nih.gov/gds). Datasets with less than seven patients or datasets containing metastatic tumors were excluded. In the included datasets, secondary tumors, metastatic tumors, normal tissue, xenografts, and cases without sex information were excluded (**Table 1**).

**Table 1:** Datasets included in the GSEA study to identify different immune process activated in men *vs*. women.

Cancer type	Dataset	N	Excluded cases	Men	Women	Platform
**Colon cancer**	GSE17538	238	6 adenomas	122	110	Affymetrix HG-U133 Plus 2
	GSE18088	53		26	27	Affymetrix HG-U133 Plus 2
**Gastric cancer**	GSE26899	108	12 GIST, 3 normal tissue	73	20	Illumina HumanHT-12 V3.0
	GSE26901	109		69	40	Illumina HumanHT-12 V3.0
**Head and neck cancer**	GSE6791	84	28 cervical tissue, 14 HN normal tissue	29	11	Affymetrix HG-U133 Plus 2
	GSE30784	229	17 dysplasia, 45 control	120	47	Affymetrix HG-U133 Plus 2
	GSE78060	30	4 normal tissue	18	8	Affymetrix HG-U133 Plus 2
	GSE34105	78	16 control	34	28	Illumina HumanHT-12 WG-DASL V4.0 R2
	GSE65858	270	14 secondary tumors	211	45	Illumina HumanHT-12 V4.0
**Liver cancer**	GSE9843	91	10 unknown sex	54	27	Affymetrix HG-U133 Plus 2
**Melanoma**	GSE15605	46		32	14	Affymetrix HG-U133 Plus 2
**Pancreas cancer**	GSE106189	35		23	14	Affymetrix HG-U133 Plus 2
**Renal cancer**	GSE11904	21		13	8	Affymetrix HG-U133A 2
	GSE36895	76	23 normal, 24 mouse	17	12	Affymetrix HG-U133 Plus 2
	GSE40435	202	101 normal	59	42	Illumina HumanHT-12 V4.0
	GSE73731	265	3 unknown sex	160	102	Affymetrix HG-U133 Plus 2
**Thyroid cancer**	GSE53157	27	3 normal	8	16	Affymetrix HG-U133 Plus 2
	GSE60542	92	34 normal, 23 lymph node metastasis	16	19	Affymetrix HG-U133 Plus 2
**Total**				1084	590	

Gene expression values were log2-transformed and median centered before analysis. In GC datasets, the “null” value was replaced by 0.000001.

### Gene Set Enrichment Analysis

The GSEA Analysis was done using the java GSEA Jar application (20), and 4,436 biological processes included in the Gene ontology version 6.2 (c5.bp.v6.2.symbols.gmt) were analyzed. Both are available at http://software.broadinstitute.org/gsea/downloads.jsp.

Patterns of immune gene sets were compared between tumors from female *vs*. male patients. We used gene set permutation without additional normalization and default parameters. The analysis was conducted individually in each dataset. A gene set was considered enriched when it was present in at least three cancer types with a p-value < 0.05.

### Gene Ontology Groups

The significantly overexpressed processes were grouped according to the Gene Ontology Browser (http://www.informatics.jax.org/vocab/gene_ontology/). The biological processes included in each group are detailed in **Supplementary Data S1**.

### Ethical Considerations

The study was approved by the Ethical Review Board of the Universidad Privada San Juan Bautista (084-2021-CIEI-UPSJB) and conducted in compliance with all relevant ethical guidelines.

## Results

### Improved Outcomes for Women in Different Cancer Types

**Figure 1:**
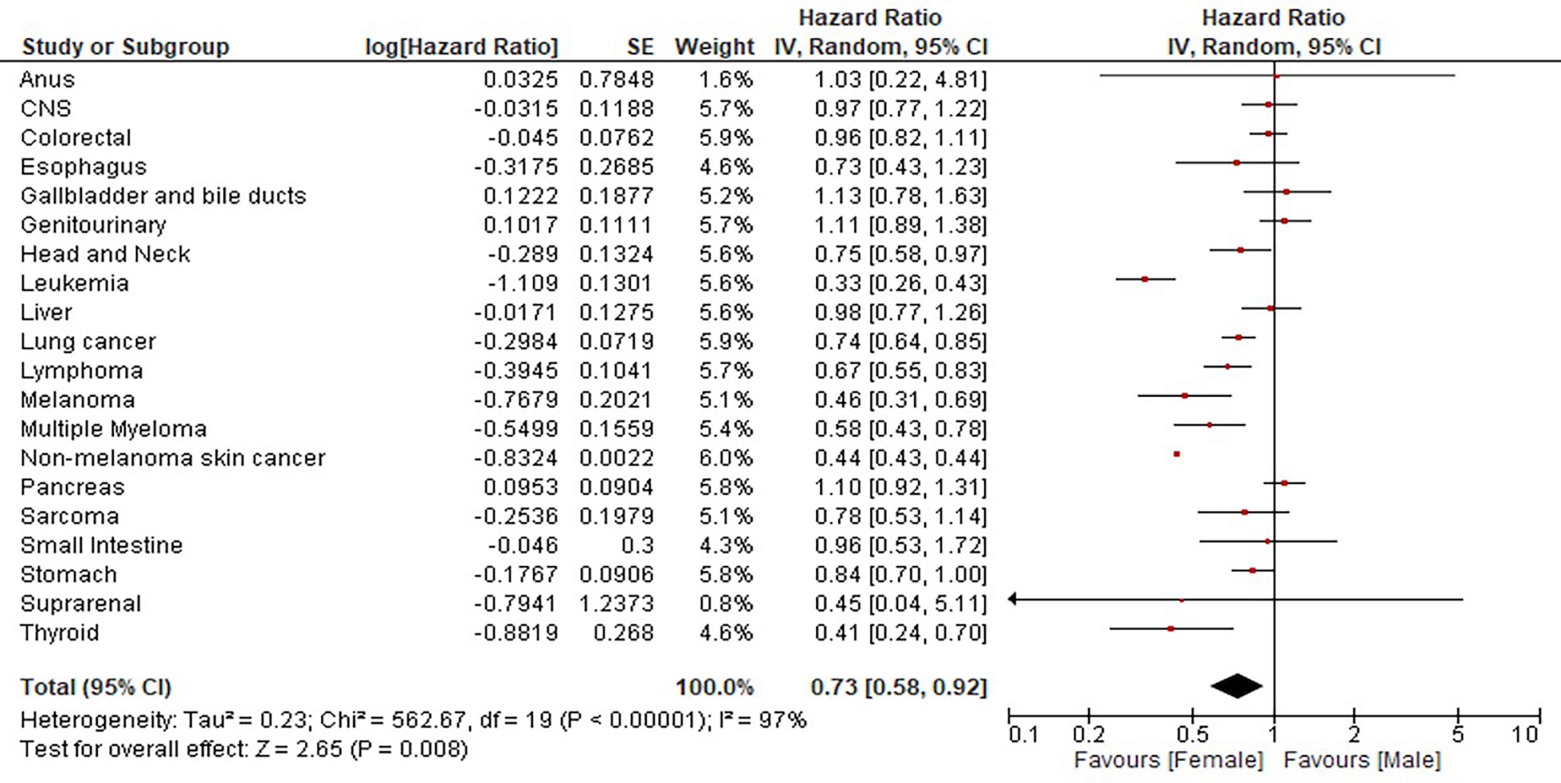
Influence of sex in the survival of 13,619 patients with 20 different cancer types.

The meta-analysis of 13,619 patients (54.4% women; 45.6% men) that pooled HR from the analysis of 20 different types of cancer determined a decreased risk of death in female patients (HR = 0.73, CI95: 0.58–0.92). Significant differences in the risk of death were observed in HNC (HR = 0.75, CI95: 0.58–0.97), leukemia (HR = 0.33, CI95: 0.26–0.43), LC (HR = 0.74, CI95: 0.64–0.85), lymphoma (HR = 0.67, CI95: 0.55–0.83), MEL (HR = 0.46, CI95: 0.31–0.69), MM (HR = 0.58, CI95: 0.43–0.78), and non-melanoma skin cancer (HR = 0.44, CI95: 0.43–0.44) (**Figure 1**).

### Differences in Mutational Status in Melanoma, Colon, and Lung Cancer

**Figure 2:**
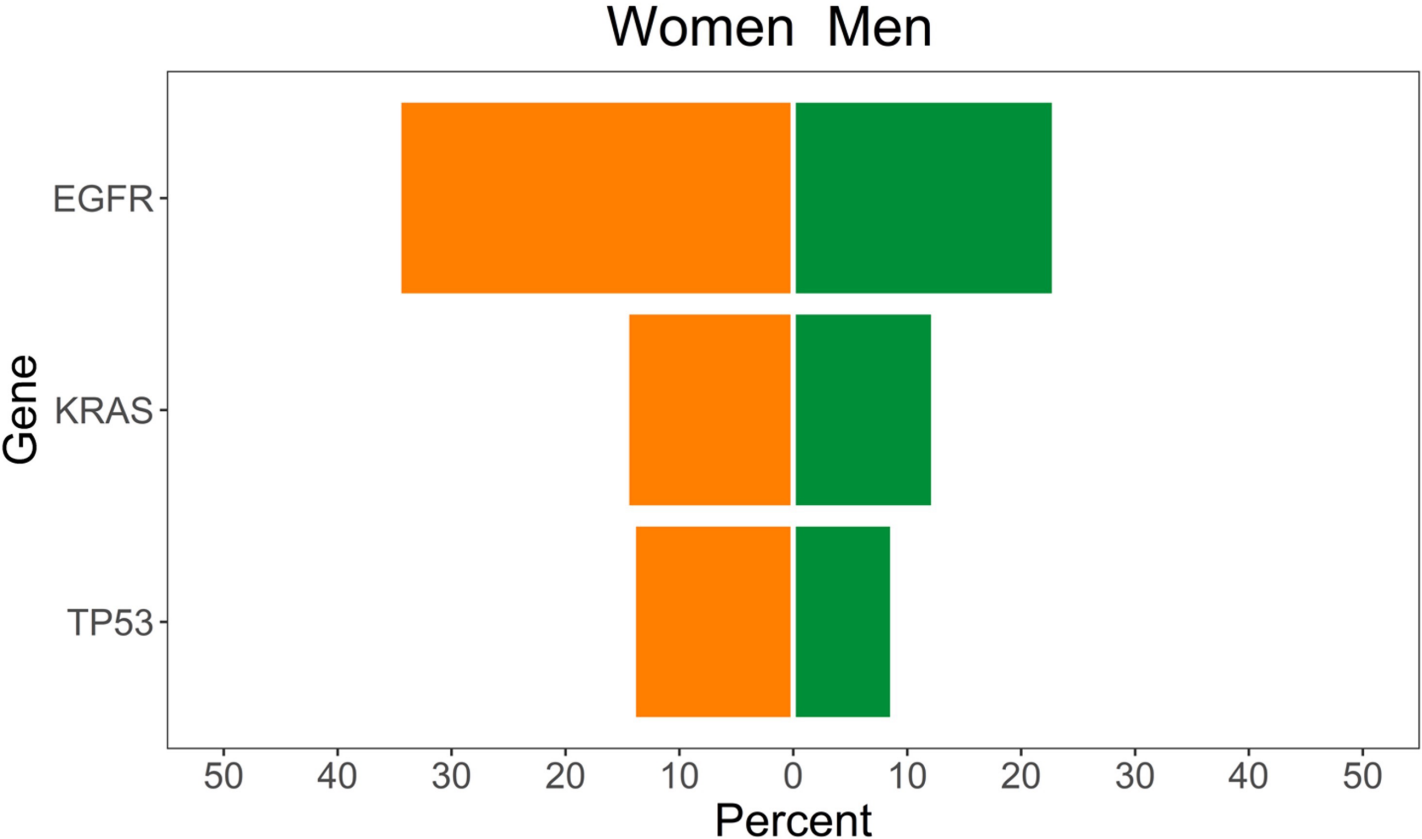
Lung cancer patients with mutations in TP53, KRAS, and EGFR, according to sex.

In total, 301 LC patients were evaluated, of which 160 were women (53.2%). A significant association between sex and mutational status was found in LC with regard to *EGFR* mutations, namely, 22.7% in men *vs*. 34.4% in women (p = 0.035) (**Figure 2**). On the contrary, the mutational status of *BRAF* in MEL (p = 0.422), *KRAS* (p = 0.341), *BRAF* (p = 0.895), and *PIK3CA* (p = 0.704) in CC patients was not related to sex (**Table 2** and **Supplementary Data S2**).

**Table 2:** Association between mutation status and sex in melanoma, colon, and lung cancer patients.

Cancer type	Gen	Status	Men		Women		p
			N	%	N	%	
Melanoma	*BRAF*	Wild type	29	55.8	34	65.4	
		Mutated	23	44.2	18	34.6	0.422
Colon	*KRAS*	Wild type	52	51.5	47	43.9	0.341
		Mutated	49	48.5	60	56.1	
	*BRAF*	Wild type	91	90.1	98	91.6	0.895
		Mutated	10	9.9	9	8.4	
	*PIK3CA*	Wild type	92	91.9	100	93.5	0.704
		Mutated	9	8.9	7	6.5	
Lung	*EGFR*	Wild type	109	77.3	105	65.6	
		Mutated	32	22.7	55	34.4	0.035*^a^*
	*KRAS*	Wild type	124	87.9	137	85.6	
		Mutated	17	12.1	23	14.4	0.674
	*TP53*	Wild type	129	91.5	138	86.2	
		Mutated	12	8.5	22	13.8	0.211

^a^Statistically significant.

### Enriched Immune Processes in Women

**Figure 3:**
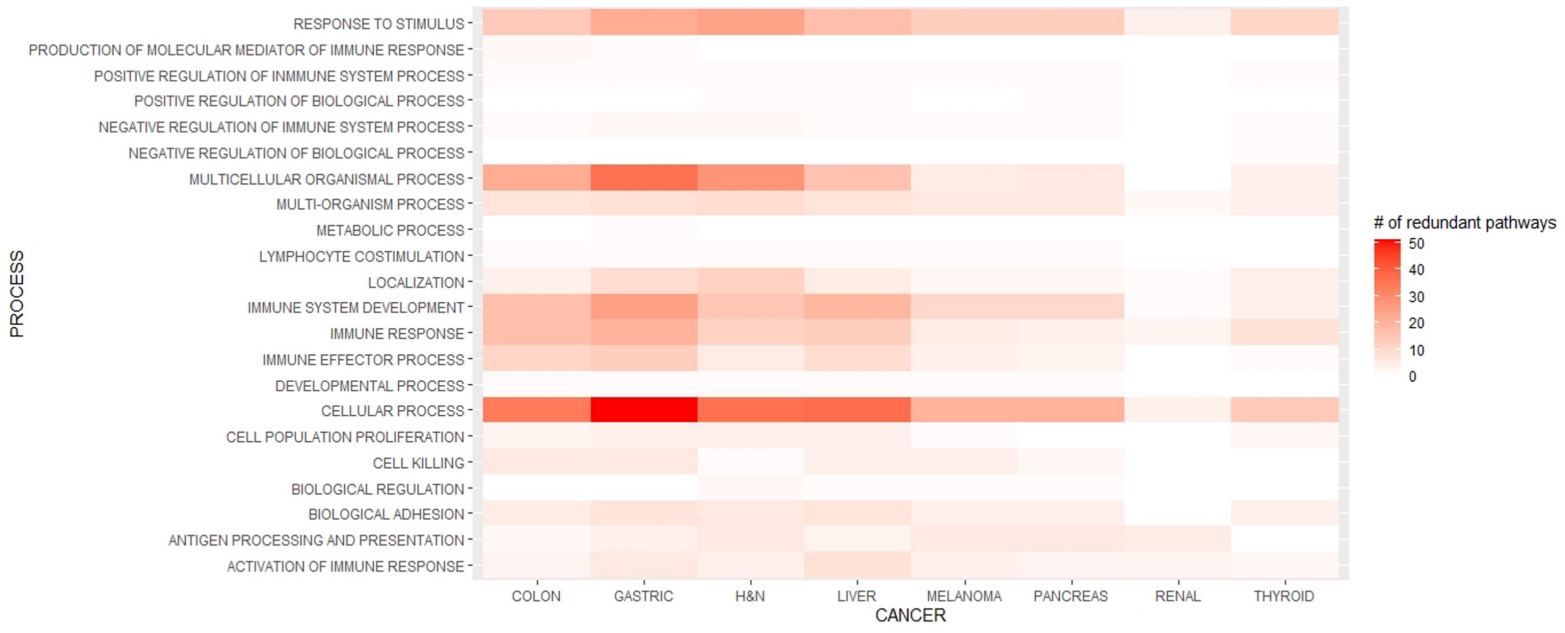
Enriched immune processes in each cancer type in women.

The number of immune processes overexpressed in women was higher than in men in GAC (218 *vs*. 48), HNC (170 *vs*. 51), LC (155 *vs*. 0), CC (151 *vs*. 3), MEL (89 *vs*. 0), and PC (85 *vs*. 0) (**Supplementary Data S1**). Overall, 22 categories of gene ontology for immune processes were enriched in tumors from women (**Figure 3**).

According to the Gene Ontology groups, the processes that were significantly enriched in women in all evaluated malignancies were related to response to stimuli, multiorganism process, localization of immune cells, immune system development, immune response, cellular processes, and activation of an immune response.

In response to stimulus pathways, the most frequent gene sets were those associated with interferon gamma (IFN-gamma), inflammatory response, positive regulation of defense response, and response to tumor necrosis factor (TNF). On the other hand, in the multiorganism process, gene sets related to innate immune response and inflammatory response were overexpressed.

In addition, processes related to the localization of immune cells included enriched expression of genes involved in leukocyte migration, lymphocyte migration, and T cell activation related to immune response.

The immune system development process was characterized by the overexpression of datasets related to lymphocyte, dendritic cell, and leukocyte differentiation; innate immune response; activating cell surface receptor signaling pathway; lymphocyte activation; positive regulation of immune effector processes; and regulation of antigen-receptor-mediated signaling pathways.

With regard to the immune response process, positive regulation of cytokine production, innate immune response, and immune cell activation as well as negative regulation of cytokine production were the most frequent gene sets.

Furthermore, in cellular processes, responses to IFN-gamma and IFN-gamma-mediated signaling pathway; cytokine-mediated signaling pathway; cell surface receptor signaling pathways; regulation of leukocyte, lymphocyte, and natural killer cell activation; Fc-gamma receptor signaling pathway; and Fc-receptor signaling pathway, among others, were overexpressed.

The most frequent gene sets in the activation of immune response processes were antigen-receptor-mediated signaling pathway, activation of immune response, and T cell-receptor signaling pathway.

### Enriched Immune Processes in Men

In total, 18 immune processes were enriched in tumors from male patients. In contrast to other cancers, in TC there was a slightly more frequent expression of IP in men compared to women (82 *vs*. 61), whereas in CRCC, the enrichment in IP was significantly increased in men (184 *vs*. 23).

**Figure 4:**
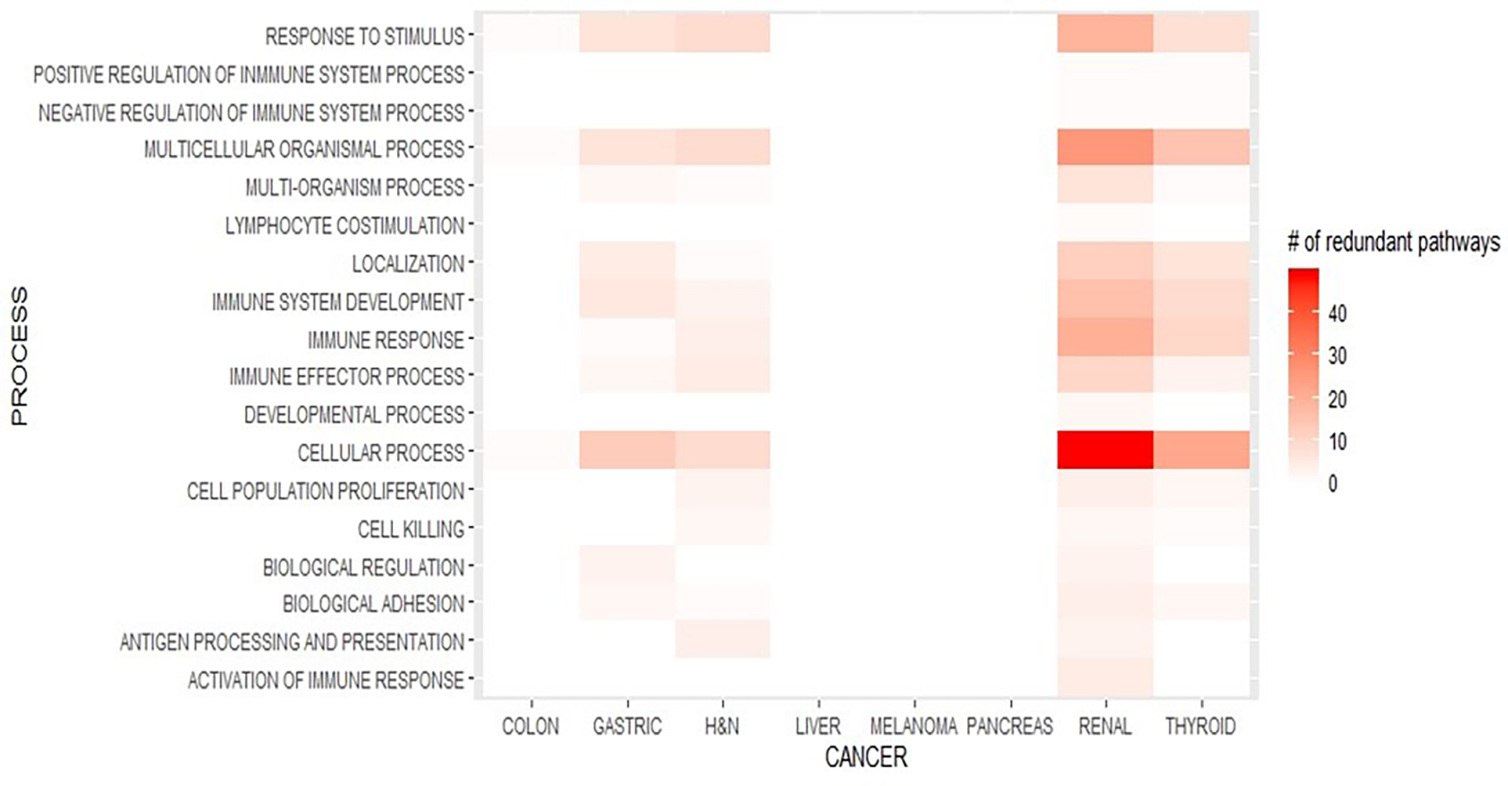
Enriched immune processes in each cancer type in men.

The most frequent gene ontology groups were cellular processes, multiorganism processes, immune response, and immune system development (**Figure 4**). Cellular processes included positive regulation of cell activation; cellular response to IFN-gamma-, chemokine-, and cytokine-mediated signaling pathways; leukocyte and lymphocyte chemotaxis; and cellular response to Interleukin 1 (IL-1). In the multiorganism process, inflammatory response, positive regulation of defense response, response to IFN-gamma, and response to bacteria were the most frequently enriched gene sets.

Regarding immune response processes, the most frequently overexpressed gene sets were regulation of cytokine secretion, positive regulation of immune response, activation of immune response, regulation of adaptive immune response, B cell-mediated immunity, and humoral immune response. In addition, in the immune system development process, positive regulation of cytokine production, regulation of T cell proliferation, and leukocyte and lymphocyte differentiation, among others, were overexpressed.

## Discussion

Cancer is one of the most important health problems worldwide, and many efforts are being conducted to improve the current therapy regimens and to develop new treatments. Within new treatments, immunotherapy has become the backbone of cancer treatment in different types of malignancies, and for this reason, there is a current race for the search of new predictive biomarkers (21).

In this study, we evaluated several features including survival and mutational status from real-world and transcriptomic datasets to explore differences between female and male cancer patients. Our study has some limitations. The hazard ratios are not adjusted for important prognostic factors in cancer such as age at diagnosis, clinical stages, and histological features, among other factors. On the other hand, the strength of our work is that we evaluated one of the largest cohorts of cancer patients in Latin America.

To explore differences in survival, we evaluated a retrospective cohort of patients admitted and followed at Oncosalud (AUNA), during the period of 2000 and 2019. Oncosalud’s pre-paid oncological plan was established in 1989 and today has close to 1 million affiliates, representing the largest cohort of this sort in Peru.

Sex-related differences in cancer outcomes are well known. According to Global Cancer Statistics, the incidence and mortality related to cancer worldwide is higher in men than in women (22). On the other hand, a study conducted with a large cohort from the Swedish Cancer Registry (n = 872,397) showed a decrease OS in men with excess mortality ratios ranging from 1.1 (CI95: 1.03–1.1) for CC to 2.1 (CI95: 1.5–2.8) in well-differentiated TC (23). In our cohort of patients, we found a 27% lower risk in death in women (8%–42%) compared to men. Analysis of sex differences in mortality in Canada determined that women had a 13% lower excess risk of death (24).

Sex might influence the effectiveness of therapy depending on the type of treatment, contributing to differences in the overall survival. Female patients with NSCLC and other cancers have better outcomes than male patients. In NSCLC, women have a 10% of additional benefit from EGFR TKI than men. In contrast, women have a reduced benefit of ICI when used as a single agent (13).

A higher activation in immune system pathways was previously shown in NSCLC, independent from smoking status or histology (14). In this work, we observed a repeated pattern of higher expression of gene sets related to immunity in women. These findings are important since the association of sex with the efficacy of immunotherapy is a complex phenomenon modeled by differences in the microenvironment. As shown by Li *et al.* (2020), treatment with atezolizumab benefits female patients (compared to chemotherapy) in terms of OS even under PD-L1 expression <1%, while male patients have no benefits (HR = 0.57; CI95: 0.38–0.85 for women *vs*. HR = 0.93; CI95: 0.68–1.26 for men) (25).

A vast majority of immune cells express receptors for estrogen and progesterone, while several immune system-related genes present elements of a response to estrogen, progesterone, and androgen receptors. These sex-related differences might therefore produce tumors evolving in different microenvironments and subsequently with different characteristics (7).

Despite the survival rates favoring female over male patients, some studies in animals have shown that estrogen has pro-metastatic activity in the liver in highly aggressive CC, pancreatic, and LC cells, mediated by the function of myeloid-derived suppressor cells and T-regs (26). On the other hand, a meta-analysis conducted by Wallis et al. (2019) suggested that there are no differences in the efficacy of immunotherapy between women and men. However, the researchers in this analysis pooled different types of malignancies or different types of ICI. In addition, they evaluated OS, not progression-free survival (PFS) (11).

In conclusion, sex is an important factor that influences the tumor microenvironment and, subsequently, the ability of the host to control the tumor, as revealed by clinical outcomes favoring female patients. A comprehensive analysis of these differences could lead to improved therapeutic strategies and discovery of new targets, particularly for immunotherapy.

## Data Availability Statement

The original contributions presented in the study are included in the article/**Supplementary Material**. Further inquiries can be directed to the corresponding author.

## Author Contributions

Study design: JA, LR, CR, MC, and JP. Data collection: JA, GR, CB-L, UI-H, KP, LG, HA, FR, SD-E, JC-F, DZ, CC-C, and MR-J. Data curation: JA, GR, CB-L, UI-H, KP, LG, HA, FR, SD-E, JC-F, DZ, CC, and MR-J. Genomic analysis: JA, GR, and CB-L. Statistical analysis: JA and JP. All authors contributed to the article and approved the submitted version.

## Funding

This study was funded by AUNA-Ideas and Oncogenomics.

## Conflict of Interest

The authors declare that the research was conducted in the absence of any commercial or financial relationships that could be construed as a potential conflict of interest.

## Publisher’s Note

All claims expressed in this article are solely those of the authors and do not necessarily represent those of their affiliated organizations, or those of the publisher, the editors and the reviewers. Any product that may be evaluated in this article, or claim that may be made by its manufacturer, is not guaranteed or endorsed by the publisher.
