# Supplementary figures and images for: Influence of Sex in the Molecular Characteristics and Outcomes of Malignant Tumors
Source: Front Oncol. 2021 Oct 19;11:752918. doi: 10.3389/fonc.2021.752918 (PMC8562721; doi:10.3389/fonc.2021.752918)

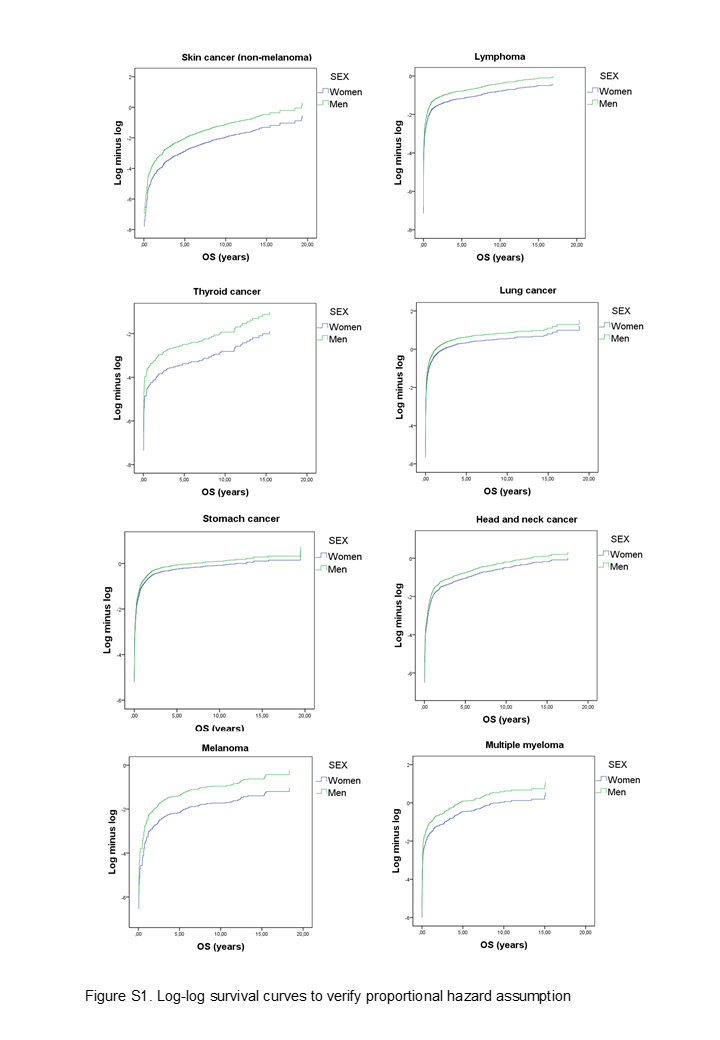

Supplement: Supplementary file 3 [file Image_1.png]
